# Preclinical evidence of polyherbal formulations on wound healing: A systematic review on research trends and perspectives

**DOI:** 10.1016/j.jaim.2023.100688

**Published:** 2023-02-24

**Authors:** Satish Dubey, Ashwini Kumar Dixit

**Affiliations:** Laboratory of Molecular Taxonomy & Medicinal Plant Biology, Department of Botany, Guru Ghasidas Vishwavidyalaya, Bilaspur 495009, Chhattisgarh, India

**Keywords:** Medicinal plants, Pharmacological activity, Polyherbal formulations, Systematic review, Wound healing, PRISMA, Preferred Reporting Items for Systematic Reviews and Meta-Analyses, CASP, Critical Appraisal skills Programme, RQ1, Research Question 1, RQ2, Research Question2, RQ3, Research Question 3, WHO, World Health Organization

## Abstract

**Background:**

The disruptions in skin integrity contribute to its disorientation, and tissue annihilations result in acute or chronic wound formation. Polyherbal formulations are widely used in traditional systems of mecdicine like ayurveda for wound healing. The combination of these traditional therapies with clinical therapies has helped in the development of various wound-healing products.

**Method:**

In this systematic review, the therapeutic potency of several polyherbal formulations from different medicinal floras is summed together in response to their impact on wound healing. The literature search was performed on Pubmed, Scopus, and ScienceDirect databases between 2010–2020. PRISMA methodology was applied to extract relevant information about polyherbal formulations.

**Result:**

A total of 54 articles were selected under all themes for the data extraction as per the PRISMA guidelines. These 54 articles have high-quality scores ≥3. Forty-three records were used for the narrative analysis, while nine records were used for the critical analysis in the narrative review. Further, theme-wise key data sets were screened from the selected literature and summarized in a tabular form. Bibliometric analysis of the Scopus database has also drawn attention to limited academic literature showcasing randomized clinical trials in the current subject. Most of these polyherbal formulations are tested in laboratory-scale studies, thus portraying further research options.

**Conclusion:**

Polyherbal formulations are effective in promoting the wound-healing process. They can stimulate a variety of physiological functions that accelerates the process of healing. These formulations merit further investigation in clinical trials, and production up scaling will aid in the creation of a new horizon of polyherbal wound healing products.

## 1 Introduction

The Indian traditional Ayurvedic system of medicine has advocated the use of natural products to promote healthy living by preventing unnecessary ailments. This traditional medical system is well-known using a variety of herbal medications to treat various causes of imbalance in human health. The fundamental principle behind the use of herbal formulations in Ayurveda is not only the restoration of a disease-free body, but also the prevention of its recurrence [1]. Herbal formulations are also common in other ancient medicinal systems, such as those of China, Egypt and GreeceAccording to World Health Organization (WHO), around 80% of people across the world still resort to these natural herbal products for the maintenance of a good lifestyle [2]. These natural products are used as a single herb or in combination with other herbs. The latter, referred to as "Polyherbal Formulations," has recently gained significant attention. The concept of “Polyherbalism” was espoused in Ayurvedic literature such as the *Sarangdhar Samhita*’ because sometimes a single herb cannot achieve the desired health effects [3]. The literature also implies that combining different herbs in an optium ratio reduces the toxicity of each herb and thus improves therapeutic effects [3]. Thus, the term "Polyherbal Formulations" refers to those pharmaceutical preparation that uses more than one herb as a component for increased therapeutic effectiveness and decreased toxicity of individual herbs.

The ancient herbal medicine system from manyparts of the world have made use of of polyherbal formulations due to their multifaceted pharmacological actions [4]. Polyherbal utilizes the concept of synergies which indicates a positive herb–herb interaction. This principle suggests that the phytochemical constituents from one plant get activated in the presence of constituents from another plant. Such interactions have been demonstrated using two mechanisms namely pharmacokinetics and pharmacodynamics. The former mechanism focuses on the facilitation of distribution, metabolism, absorption, and elimination of one herbal constituent by another. However, the latter mechanism focuses on the synergistic effect of one herb with other, when the phytochemical constituents have similar therapeutic activities and thus when targeted to a similar receptor enhances the overall pharmacological effect. It also advocates the fact that the use of multiple herbs in a single formulation may help in targeting many potential targets in the physiological system at the same time imparting faster relief. Therefore both these mechanism supports the use of multiple herbs rather than single herbal formulation. Medical practitioners also believe that polyherbal formulations help in lowering the dose size for each herbal constituent, reducing the risk of any side effect due to high dose of herbs [5]. Other than this, polyherbal formulations make it easy for patients to consume these herbs by nullifying the need to consumemany medicines separately. This improves compliance along with improved pharmacological effects.

Contemporary science has proven the effectiveness of these polyherbal formulations in the treatment of various ailments. The therapeutic potential of these Polyherbal formulations have been demonstrated against various acute and chronic diseases such as diabetes, wound care, hypertension, cardiovascular disorders, anxiety, neurological imbalances, disorders related to the gastrointestinal tract, respiratory tract, and endocrine system. Out of many applications of polyherbal formulations, the wound healing potential is the most recognized one. Traditional healing agents have been significant in wound care since ancient times. The combination of these traditional therapies with clinical therapies has helped in the development of various wound healing products with greater efficiencies.

Therefore the current review is a systematic compilation of various Polyherbal formulations tested on different models for investigating their wound healing potentials during the last decade.

RQ1 Which polyherbal formulations have shown great preclinical prevalence of wound healing?

The review also focuses on two important pharmacological aspect of Polyherbal formulations namely anti inflammatory effect and antimicrobial effect which makes a great significance in wound healing process.

RQ2 Which polyherbal formulation aids wound healing by anti inflammatory effect?

RQ3 Which polyherbal formulation aids wound healing by anti bacterial effect?

The review also makes an attempt to present the bibliometric trend of research and development in various dimensions concerning application of polyherbal formulations in wound healing.

Thus, the major aim of this systematic review is to report recently published primary literature related to wound healing and polyherbal formulations in order to trace the gaps and develop a vivid future outlook.

## 2 Research methodology

The current literature review adapts the review method given by Webster and Watson, 2002 called concept driven systematic review approach [6]. This review method examines the literature from the concept perspective of various authors. It is different from an author-driven approach that exclusively looks into the analysis of individual authors for multiple concepts in articles. As the literature on polyherbal formulation is vast and extensive, the former method is suitable for review in the current topic. In recent times, the popularity of polyherbal formulations has drawn the attention of various researchers on this topic, thus polyherbalism seems to be a major emerging area in alternative medicine. Therefore this method of review helps capture the related studies in an easy and concise manner.

### 2.1 Sources

The review process was initiated by looking at three major research databases, SCOPUS, PubMed, and ScienceDirect to collect related articles. However, essentially every article that was located in the ScienceDirect and PubMed databases was also available in the Scopus database. For this reason, the SCOPUS database was selected as the source for gathering primary data for the review. The articles were selected from the database using relevant keywords like “Polyherbal formulation”, “Polyherbalism”, “Herbal medicines”, “Herbal therapy”, “Traditional medicines”, “Ayurvedic medicine” etc. These collected research papers were inspected thoroughly for further analysis.

### 2.2 Theme identification

The selection of theme for the review was done after a stringent analysis of the research papers retrieved under the aforementioned keywords. The analysis was done using a research framework that first focused on title screening. This was undertaken to identify major themes under the research topic in terms of the application of the polyherbal formulations as mentioned in the papers. This led to the categorization of these papers under various research themes. The theme that was identified to have a major contribution to the literature was chosen for the review. The major theme was further screened for specific sub-themes.

### 2.3 Data extraction and synthesis

Following the identification of a major theme, the papers chosen under the sub-themes were submitted to comprehensive reading. The quality assessment of the selected paper in each sub-theme was done by the two reviewers (Author 1 and Author 2) using a modified version of the Critical Appraisals Skills Program (CASP) quality assessment tool for randomized control trials and cohort studies (Supplementary Item 1). The review team used the most relevant items from the checklist to establish the quality parameters. Following an independent evaluation of the full text papers, a detailed spreadsheet was created, and the quality scoring by both reviewers was recorded. The scoring was done on a scale of 5 and the average score allocated by the reviewers has been considered the final quality score for each paper. A third reviewer was consulted in case the first two reviewers could not reach a particular consensus. Only those reports having a high quality score (≥3) have been selected for data extraction. To prevent bias in selection of papers responses from each author were kept blinded from each other. The Preferred Reporting Items for Systematic Review and Meta-Analysis (PRISMA) flow chart has then been prepared demarcating the inclusion criteria for the selected papers.

The SCOPUS database was also used to obtain various bibliometric trends which were used to analyze various perceptive of research contributions under this research topic.

## 3 Results

Database searches could retrieve 7481 research papers using the keywords above. After removing duplicates and papers published before 2010, approximately 973 were finally subjected to title screening to retrieve the major theme. The database search depicted many applications of polyherbal formulations like wound healing, anti-cancer, immunostimulants, or as behavioral and neurochemical drugs. Of all these applications, around 574 papers were related to the wound-healing properties of various polyherbal formulations. Thus, “polyherbal formulations in wound healing” was chosen as the central theme for the review. Two minor themes related to wound healing could also be identified during the literature review. Therefore the three themes identified and reviewed in the current article are stated below:

Theme 1: Polyherbal as wound care formulations.

Theme2: Polyherbal formulations promoting anti-inflammatory activities aiding wound healing.

Theme 3: Polyherbal formulations against infections aiding wound healing.

**Fig. 1:**
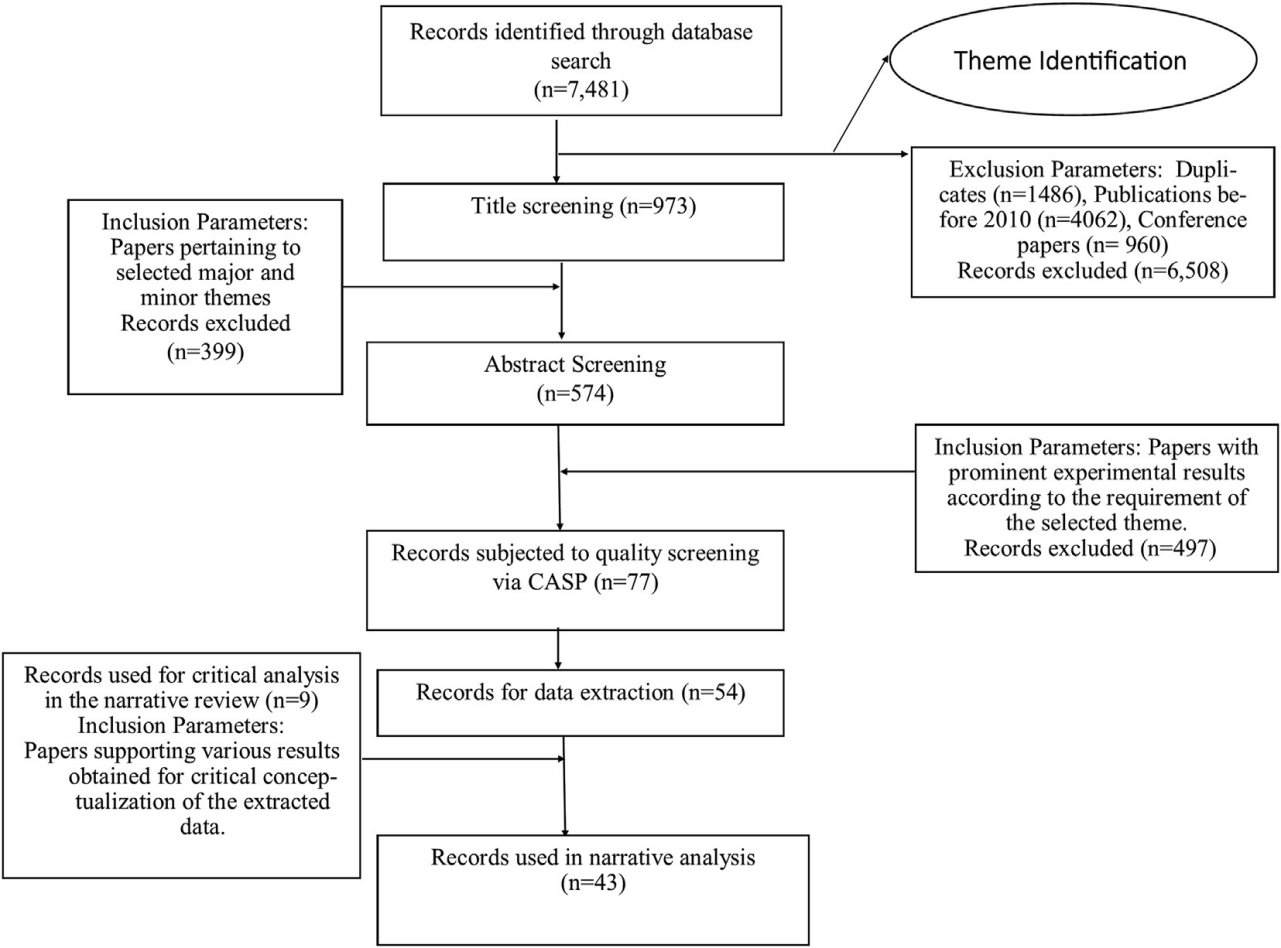
PRISMA (Preferred Reporting Items for Systematic Reviews and Meta-Analyses) Scheme for selection of articles for the systematic review.

**Fig. 2:**
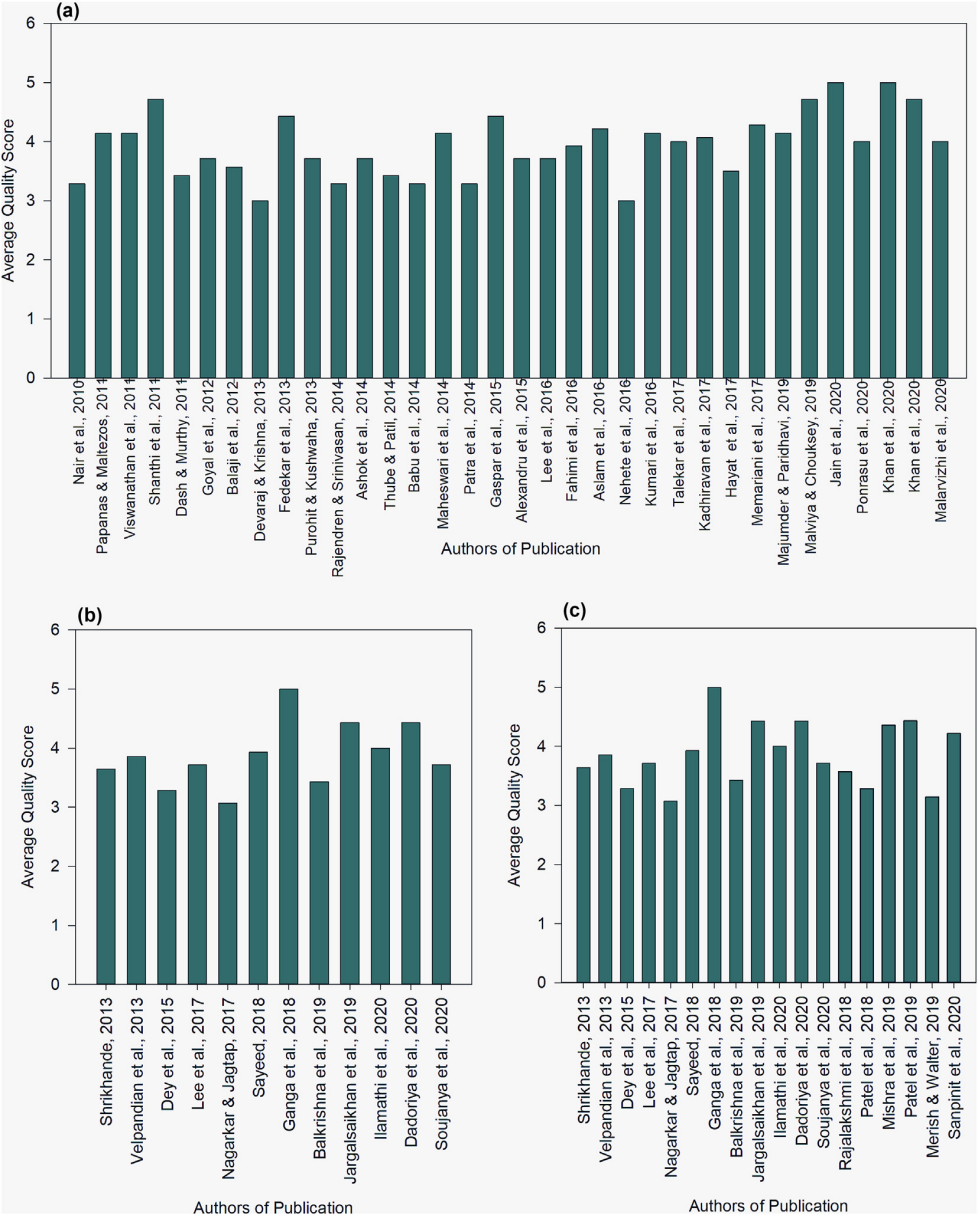
Quality Score Analysis of the reviewed records under theme 1 (a), theme 2 (b), and theme 3 (c) using modified CASP checklist.

After screening the abstracts, 742 articles were selected for full-text screening through critical appraisal by both the reviewers (Fig. 1). The average quality scoring obtained for papers under all the aforementioned themes after critical appraisal by both the reviewers gave 54 articles having a final quality score equal to or above 3 (Fig. 2).

For the data extraction, the following key data were screened from the selected literature: details of the authors; year of publication; herbal medicinal product; study model; tested pharmacological implication of the polyherbal product, and special highlight in results (if any). These key data sets were summarized in a tabular format to ease further theme wise analysis (Table 1):

**Table 1:** Data Extraction for the quality papers selected for review under the identified themes.

S.No.	Polyherbal Formulation Composition	Name of Formulations	Testing Model (if any)	Pharmacological Activities of Formulations	Author's name
**Theme1: Polyherbal as wound care formulations**					
1.	*Allium sativum* (bulb) *Zingiber officinale* (rhizome) and *Curcuma longa* (Rhizome)	Aab-e-Shifa	Rabbits	Wound healing associated with phytochemical constituents of all the herbs	[7]
2.	Natural honey, Olive oil and Beeswax	Derma heal cream	Rats	Effective in wound and burn healing in both normal and diabetic rats	[8]
3.	*Guggalsalai, Shtavar* (leaf), *Nirugundi* (leaf), *Karpor oil, Ashwagandha* (leaf)	Amrit oil	Wistar Rat	Effective against excision wound	[9]
4.	*Euphorbia hirta Linn., Tridax procumbens* and *Eclipta alba*	–	Albino rats by using three	Effective in healing three wound models *i.e.* excision wound, incision wound and dead space wound models	[10]
5.	Salicylic acid, Green tea extract, *Phyllanthus emblica* and *Zingiber officinale Rosc*		Male foreskin fibroblasts (HS68)	Promotes wound contractionand promote re-epithelialization in skin tissue engineering by the extracellular matrix properties	[11]
6.	*Cassia auriculata, Mangifera indica, Ficus banghalensis, Cinnamomum tamala* and *Trichosynthis diocia*		Rats	Effective in healing three wound models i.e. excision wound, incision wound and dead space wound models	[12]
7.	*Plumbago zeylanica Linn, Datura stramonium* Linn and *Argemone mexicana* Linn	Polyherbal carbopol- 940 gels	Wistar albino rat	Antimicrobial, anti-inflammatory accelerated tissue remodeling	[13]
8.	Lablab purpureous, *Nerium indicum*, Tebernamontana derivitacate	–	Wister rat in excision and incision wound model	Promoted rapid wound contraction	[14]
9	*Vitex negundo* L.*, Emblica officinalis Gaertn,* and *Tridax procumbens*	–	L929 fibroblastic cell line and HaCaT keratinocytes cell line	Promotion of regeneration of skin, wound contraction and collagen synthesis at the site of injury in excision wound model.	[15]
10.	*Cordial oblique* Willd*., Dentophote falcate ettingsh., and Vigna radiate* Linn*.*	Polyherbal ointment	Albino rats	Promotes wound contraction	[16]
11.	*Bambusa arundinacea, Coriandrum sativum, Elettria cardamomum, Foeniculum vulgare, Rosa damascene, Mineral bezoar triturated* and *Pistacia lentiscus.*	Ulcerene	Male rabbits and albino rats	ethanol, aspirin and stress-induced gastric ulcer model of rat against peptic ulcers	[17]
12	*Rosa damascena* mill. (Petals), *Glycyrrhiza glabra* L. (Rhizomes) and *Nardostachys jatamansi* DC.	VARD	Wistar male rats	Effective against gastric ulcers partially via antioxidant activity	[18]
13.	*Ostericum koreanum rhizome, Anglicae gigas* (root), *Ledebouriella seseloides* (root), *Paeonia Lactiflora* (root), *Rehmannia glutinosa* (root), and *Cnidium officinale Makino*	Bo-Gan-Whan	Rats	Anti-atherosclerosis effect: Inhibits proliferation and migration of PDGF-BB-stimulated Vascular Smooth Muscle Cells thus promotes wound healing	[19]
14.	*Malva sylvestris, Rosa damascene, Solanum nigrum*	Iranian Traditional Medicine (ITM)	–	Burn healing	[20]
15.	*Elephanto pusscaber* and *Clinacanthus nutans*	–	Swiss albino mice	Effective in wound healing for excision wound model, incision wound model, and burn wound model	[21]
16.	*Ari's Wound Healing Cream: Glycyrrhiza glabra, Ficus infectoria, Shorea robusta, Curcuma longa, Berberis aristata, Rubia cordifolia, Azadirachta indica, Pongamia glabra,* and *Yashad Bhasma* *Amarantha Wound Healing Cream: Jatyadi Oil, Yashad Bhasma, Ficus religiosa, Ficus bengalensis, Centella asiatica, S. robusta, G. glabra, A. indica, and P. glabra.*	Ari's Wound Healing Cream and Amarantha Wound Healing Cream	Both sexes of albino wistar rats	Both the creams were effective for faster healing of diabetic wound model in rats by enhanced epithelialization and cellular proliferation.	[22]
17.	*Glycyrrhiza glabra, Emblica officinalis* and *Morinda citrifolia*	–	Wistar male albino rats	Effective in treatment of Gastric Ulcers	[23]
18.	*Equisetum arvense, Achillea millefolium, Echinacea purpurea, Hyssopus officinalis*	–	L929 fibroblast cell culture	Promotes collagen synthesis thus aids wound healing	[24]
19.	*Achillea millefolium* L.*, Hyssopus officinalis* L.*, Equisetum arvense* L. and *Echinacea purpurea* L.	–	L929 fibroblast cell culture	Promotes enhanced collagen synthesis	[25]
20.	*-*	RhanaThaila (Siddha Formulation)	Albino wister rats	Excision wound healing model	[26]
21.	GC01: *Butea monosperma* Lam*., Symplocos racemosa* Roxb*., Mimosa pudica* Linn*., Curcuma longa* Linn*., Commiphora mukul, Piper longum* Linn*., Azadarchta indica* Linn*., Pongamia glabra, Cocos nucifera* Linn*.* and Ghrita (Ghee) GC02: *Myristica fragrans Houtt., Melia azadarch* Linn*., Trichosanthes cucumerina* Linn*., Picrorhiza kurroa,* *Berberis aristata* DC*., Curcuma longa* Linn*., Hemidesmus indicus* R*.,* *Rubia cordifolia* Linn*., Terminalia chebula, Vetiveria zizanioides* (L.), *Madhuca longifolia* and *Pongamia glabra*	GC01, GC02	Rats	synergistic/potentiative action was observed for individual herb	[27]
22.	*Butea monosperma, Barleria prionitis, Casuarina equisetifolia, Dalbergia sissoo,* and *Lagenaria siceraria*	–	Sprague Dawley rats	Excision and incision wound model faster wound size reduction and wound closure rates	[28]
23.	*Ixora coccinea* (flowers)*, Psidium guajava* (roots), *Neolamarckia cadamba* (leaves).	–	Wistar male albino rats	Anti-ulcer quality by virtue of antioxidant nature	[29]
24.	*Ficus glomerata Roxb.* (Bark);, *Fagonia Arabica* L. (whole plant), *Vetiveria zizanioides Stapf* (root)*, Santalum album* L. (wood), *Andrographis paniculata Nees* (whole plant), *Melia azadirachta* L., (bark), *Terminalia chebula* Retz (bark)*, Terminalia bellerica* Roxb (bark)*; Emblica officinalis Gaertn* (fruit), *Trichosanthes dioica Wall* (whole plant)*, Adhatoda vasica Nees* (leaf), *Tinospora cordifolia Miers* (whole plant)*, Fumaria officinalis* L*.* (whole plant)*; Shauktik bhasma, Kapardika bhasma, Praval bhasma*	Normacid syrup	Swiss albino mice	Effective in treatment of Peptic ulcer disease by virtue of antioxidant nature	[30]
25.	*Withania somnifera, Zingiber officinale, Piper Longum, Piper Nigrum, Elettaria cardamomum, Cinnamomum verum, Syzygium aromaticum, Saccharum officinarum,*	Amukkara choornam	Albino rats	Possesses anti-ulcerogenic activity supported by free radical scavenging activities and antihistaminic-like effects.	[31]
26.	*Moringa oleifera, Raphinus sativus,* and *Amaranthus tricolor*	–	Albino rats	Antiulcer genic activity by enhancing mucus secretion and prostaglandins	[32]
27.	*Glycyrrhiza glabra, Garcinia cambogia, deglycyrrhizinated licorice* extract and *Azadirachta indica.*	–	Rodents	Gastric wound healing property by virtue of free radical scavenging activity.	[33]
28.	*Allium sativum, Zingiber officinale, Liquorice, Cystoseira trinoddis*	–	Diabetes induced Rats	Reduction in wound closure time, faster wound healing and contraction	[34]
29.	*Calotropis gigentea* Linn*.* (Root Bark)*, Nyctanthes arbor-tristis* Linn*.* (Leaves)*,* and *Tridax procumbens* (flower)	–	Rats	Greater collagen synthesis	[35]
30.	*Glycyrrhiza glabra, Terminalia chebula, Zingiber officinale, Cassia senna, Operculina turpethum, Asparagus racemosus, Aloe barbadensis*	–	Wistarrats	Anti-ulcerogenic activity by free radicals scavenging activity and thus decreasing lipid peroxidation	[36]
31.	*Ageratum conyzoides* Linn*., Argemone Mexicana* Linn*., Heliotropium indicum* Linn*. A*nd *Alstonia scholaris (*L*.) R. Brown.* (Bark)	–	Rats	Enhanced rate of wound closure and faster epithelialization	[37]
32.	*Glycyrrhiza glabra, Musa paradisiaca, Curcuma longa, Pandanusoda ratissimus, Aloe vera, Cocos nucifera oil*	–	Diabetic foot ulcers	Also depicts anti-infectious nature	[38]
33.	*Glycyrrhiza glabra, Musa paradisiaca, Curcuma longa, Pandanusoda ratissimus, Aloe vera, Cocos nucifera oil*	–	Diabetic foot ulcers	Anti-ulcergenic activity	[39]
34.	*Glycyrrhizia glabra* (Rhizome)*, Aegle marmelos* (leaf), *Hemidesmus indicus* and *Cuminum cyminum* (fruit)	–	Wistar rats	Ulcer healing activity by prevention of mucosal lesions and sub-mucosal oedema	[40]
35.	*Withania somnifera* (Root), *Ocimum sanctum* (leaves)*, Camellia sinensis,* (leaves), Triphala*, and* shilajit	NR-ANX-C	Rats	Anti-ulcer activity by decreasing lipid peroxidation	[41]
**Theme 2: Polyherbal formulations promoting anti-inflammatory activities aiding wound healing**					
36.	*Ocimum basilicum* L*.,* *Curcuma longa* L.*, Citrus lemon* L.*, Allium sativum* L.*, Plectranthus amboinicus* Lour	Vipro™	Wistar rats	Anti-inflammatory in naturedecreases TNF-α level.	[42]
37.	*Acacia nilotica* (Stem bark)*, Withania somnifera* (Roots)*, Juniperus communis* (fruits)*, Tinospora cordifolia* (stem)*, Aspargus recemosus, (Gokshuara* roots) *Tribulus terrestris,* (fruits)*, Argyeria nervosa* (roots)*, Pluchea lanceolata* (roots & leaves)*, Anethum sowa* (fruit)*, Hidichium spicatum* (rhizome)*, Trachyspermum ammi* (fruits)*, Zingibero fficinale, shuddha* (Rhizome)*, Commiphora mukul* and *ghee* (exudates)	Trayodashangguggulu	In-vito studies	*In-vito* anti-inflammatory activities by the virtue of anti-lipoxygenase and anti-proteinase activities	[43]
38.	*Zingiber officinale, Curcuma longa, Aloe barbadensis, Citrus aurantium, Emblica officinalis* and castor oil	–	Rats	Anti-inflammatory activity accelerated excision wound healing process	[44]
39.	*Balsamodendron mukul, Colchicum luteum, Withania somnifera, Asphaltum, Strychnos nuxvomica, Cyperuss cariosus, Pluchea lanceolata, Vitex negundo, Boerhaavia diffusa, Trigonella foenumgraecum, Operculina turpethum, Asparagus racemosus, Cissus quadrangularis, Curcuma longa, Zingiber officinale, Picrorhiza kurroa, Godantibhasma, Muktashuktibhasma, Yograg guggul* (Classical product)*, Trachyspermum ammi, Corallium rubrum, Vitex nigundo, Dasmool* (Classical product)*, Tinospora cordifolia)* and *Gum acacia*	PeedantakVati	Male Wistar rats	Anti-inflammatory and analgesic property by inhibiting pro inflammatory cytokinin *viz*. decrease in IL-6 and TNF-α	[45]
40	*Artemisia santolinifolia* Turcz*, Saussurea salicifolia* L*.* and *Hippophae rhamnoides* L*.*	–	Male adult wistar rats	Anti-inflammatory activity by significant decrease inpro-inflammatory cytokine IL-1β, TNF-α and HMGB-1	[46]
41	*Azadirecta indica*, *Tinospora cordifolia, Tricosanthus cucumerina*, *Solanum xanthocarpum*, *Bambusa arundinaccea*, *Emblica officinalis*, *Terminalia belerica*, *Terminalia chebula*, *Zinziber officinale*, *Piper nigrum*, *Piper longum, Cyperus rotandus, Curcuma longa*, *Berberis aristata*, *Holarhenaanti dysentrica*	Guggulutiktakaghritam	*In-vitro* study using monocytes as model	Anti-inflammatory effect by modulation of Pro-inflammatory cytokines TNF-α and IL-1β	[47]
42.	*Colchicum luteum, Butea frondosa, Withania somnifera. Pyrethrum indicum, Myrtuscaryophyllus Zinziber officinalis* and *Allium cape*	–	Albino rats with Paw Edema	Anti-inflammatory	[48]
43.	*Zingiber officinalis* (Rhizome)*, Piper nigrum* (Fruit), *Calotropis gigantean* (flower), *Bans Bambusa* (leaf)	HabbeGuleAakh	Wistar rats and Swiss mice with Paw Edema	Anti-inflammatory and pain reliever	[49]
44.	*Paeonia suffruticosa* Andr., *Prunus persica* L., *Trichosanthes kirilowii* Maxim, *Rheum plamatum* L., Mirabilite	Daehwangmokdantang (DHMDT)	Murine macrophage-like RAW 264.7 cells	Anti-inflammatory in nature by suppression of LPS-induced phosphorylation of Akt and MAPKs in RAW 264.7 macrophages	[50]
45.	*Aeglemarmelos,* (Root), *Asparagus racemosus (*Root), *Curcuma longa* (Rhizomes), *Desmodiumgangeticum* (Root), *Gmelina arborea* (Root), *Oroxylum indicum* (Root), *Pongamia pinnata* (L.) (Stem bark), *Premna obtusifolia* (Root) *Solanum anguivi* (Root), *Solanum virginianum* (Root), *Stereospermum colais* (Root), *Terminalia chebula* (Fruit), *Tribulus terrestris* (Root), *Uraria picta* (Root)	DF1911, DF2112 and DF2813	Female Wistar albino rats with Paw Edema	Higher Anti-inflammatory effects than Dashamoola Kwatha	[51]
46.	*Withania somnifera, Boswellia serrata, Zingiber officinale,* and *Curcuma longa,*	BV-9238	Sprague Dawley rats	Anti-inflammatory	[52]
47.	Tea tree oil, Lemongrass oil, Ginger oleoresin & Capsaicin and Cow Ghee	Polyherbal emulgel	Male wistar albino rats	Anti-inflammatory	[53]
48.	*Azadirachta indica, Moringa pterygosperma, Eclipta alba, Boerhaavia diffusa, Carumcopticum, Terminalia chebula, Terminalia belerica, Emblica officinalis, Santalum album, Ocimum sanctum, Vitex negundo, Curcuma longa, Mentha piperata, Cinnamomum camphora, Amomum subulatum, Rosa centifolia,* Rock salt, Pearl, Honey	Itone™	Wistar albino rats	Anti-inflammatory activity by reduction of LTB4 formation	[54]
**Theme 3: Polyherbal formulations against infections aiding wound healing**					
49.	*Garcinia mangostana*, *Oryza sativa*, *Curcuma longa*, and *Areca catechu*	–	Human (Diabetic foot ulcers)	Antibacterial activity against *Staphylococcus epidermidis* and *Pseudomonas aeruginosa*	[55]
50.	Acacia *catechu* (heart wood), *Lagerstroemia* *speciosa* (Leaves), *Aegle marmelos* (fruits), *Phyllanthus emblica* (fruits) and *Terminalia chebula* (fruits)	Polyherbal Gel	Human vaginal keratinocyte cell line	Anti-HIV-1 activity and also effective in healing vaginal wounds caused by *Lactobacillus* infection	[56]
51.	*Glycyrrhiza glabra, Santalum album, Piper longum, Hemidesmus indicus,* Kadugarogini*, Syzygium aromaticum, Vettiveria zizanoides Plectranthus vettiveroides* (Vilamichuver)*, Nymphaea pubesens*	Sagadevinei	*In-vitro*	Antibacterial (Inhibits *E-Coli* biofilm formation)	[57]
52.	*Azadirachta indica* (Leaves), *Acacia nilotica* (Bark), *Ocimum sanctum* (Leaves), *Annona squamosal* (Leaves), *Curcuma longa* (Rhizome 7%), *Ricinus communis* (Seed oil), Beewax	Herboheal	*In-vitro* and *in-vivo* (*Caenorhabditis elegans*as model)	Effective against wound infective *C. violaceum* and *S. marcescens*	[58]
53.	*Cassia auriculata, Cassia fistula, Syzygium jambos, Olaxscandens, Saussurea lappa, Terminalia arjuna, Cyperus rotundus*	AaviraiKudineer	*In-vitro*	Antibacterial activity *Bacillus* species that causes infections in wounds.	[59]
54.	*Azadirachta indica* (Leaves), *Acacia nilotica* (Bark), *Ocimum sanctum* (Leaves), *Annona squamosal* (Leaves), *Curcuma longa* (Rhizome 7%), *Ricinus communis* (Seed oil), Beewax	Herboheal	*In-vitro* and *in-vivo* (*Caenorhabditis elegans*as model)	Antibacterial activity against wound infective *S. aureus*	[60]

## 4 Discussion

Human skin has an innate ability to regenerate after injury or damage. However, a number of underlying factors, such as diabetes, severe burns and substantial skin loss, frequently impede the process of self-regeneration process [61,62]. This leads to wounds which are difficult to be self cured. Such wounds become hosts for microbial colonies, greatly increasing the risk of infection [63]. These wounds not only cause pain but they also negatively impact their general health and cause related social issues. Inappropriate healing conditions may necessitate the use of expensive and sophisticated wound care products, as well as extended period of hospitalization. Therefore contemporary research in alternative medicines has made efforts to figure out traditional therapies that aid in wound-healing.

The current review has reported a combination of many herbal compounds showing effective wound healing potential. Many authors in this context have reported the phytochemical composition of the poly-herbal compounds in relation to the wound healing potentials [7,15,17,22,25,29,35]. In this connection, many authors have reported secondary metabolites having an antioxidant nature, such as flavonoids and active phenol compounds present to be responsible for the wound healing process [8,18,24,29,71].

Several medicinal plants are widely used as the active compound in various polyherbal formulations. Plants like *Aloe vera* contain many natural bioactive compounds like anthraquinones, saponins, and pyrocatechol making it a potent antimicrobial agent [64]. *Arctium lappa* was found to have elevated dermal ECM metabolism reducing wrinkles in human skin in vivo [65]. Formulations of *Astragalus propinquus* and *Rehmannia glutinosa* roots are widely used in diabetic wound healing [66]. *Ampelopsis japonica* is actively used in the treatment of ulcers [67]. *Calendula officinalis* is also used in many polyherbal formulations for the treatment of wounds [68].

Many studies have suggested various targets for these active compounds that ultimately help to heal. These are mediated by multiple cascades, which includes mitogenic pathways [22,24,25,50], extracellular matrix synthesis pathway [11], free radical scavenging pathway [13,[29], [30], [31],^33^,^36^,^41^], atherosclerosis pathway [19] and anti-inflammatory pathways [48,49,51]. It is therefore evident that the majorly studied pathway from the aforementioned is the free radical scavenging pathway. Therefore, formulations with high antioxidant potential are good healing agents [69]. This may be because of the fact that antioxidants scavenge the free radicals, thereby controlling oxidative stress and accelerating the healing process [70].

**Fig. 3:**
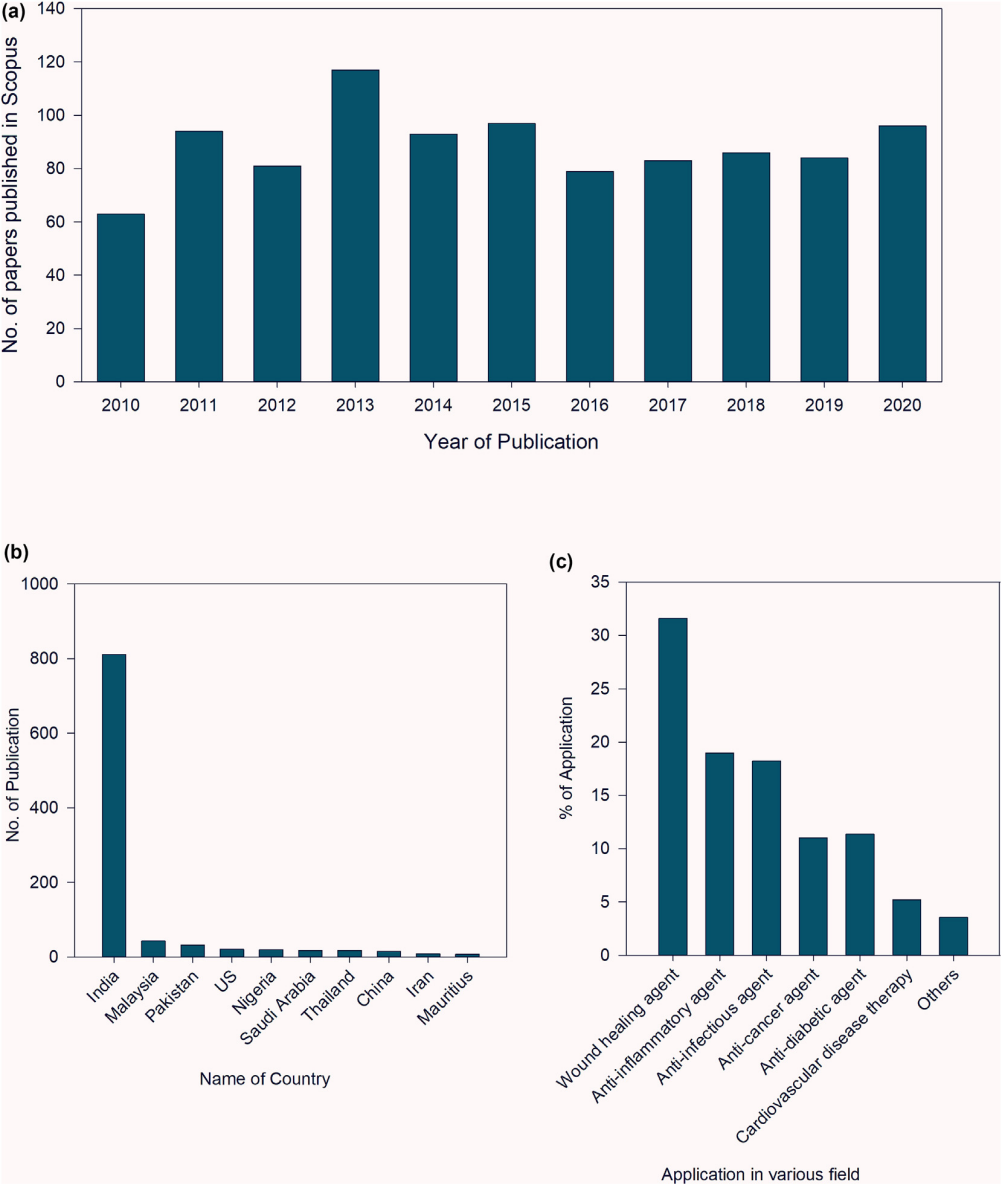
Trend of research in the field of polyherbal formulation (a) Bibliometric trend in publications records in polyherbal formulations from 2010 to 2020 in SCOPUS database (b) Country wise bibliometric trend in publications data in polyherbal formulations 2010–2020 in SCOPUS database (c) Relative abundance of various applications of polyherbal formulations investigated in the existing studies (SCOPUS database).

The literature review depicts that many polyherbal formulations have been reported to enhance numerous processes during wound healing. These processes majorly involve epithelialization, collagen synthesis and wound contraction. This could also be evaluated from bibliometric analysis of the SCOPUS database for research on polyherbal formulations in wound healing. The increasing trend of academic research in this field indicates the growing attention to research in Polyherbal medicine (Fig. 3a). Interestingly, most of these research papers concentrate on India (Fig. 3b), and different aspect of wound healing are the most studied application of polyherbal medicines (Fig. 3c).

Despite extensive research in the subject of wound healing, it is challenging to demonstrate the effectiveness of polyherbal formulations as wound healing agents. This is because there were relatively few randomised clinical trials and the majority of research were restricted to in vivo lab scale studies. It is challenging to view the potentials these formulations displayed in in-vivo lab research as their ultimate prospects. The wound models used in most cases are freshly prepared and free from microbial load. However, in actual conditions, the growth of microbial colonies or deposition of microbial products on an open wound is very common, ultimately slows down the rate of wound healing. Few studies in this context have demonstrated the antimicrobial nature of the polyherbal formulations meant for wound healing purposes. However, these studies are only limited to a particular cell line or are in vitro studies. As healing is not just limited to uninfected wounds, to achieve meaningful and concrete conclusions it is recommended that the studies concerning polyherbal formulations as wound healing agents need practical protocols with clinical situations.

Data from the earlier studies depicts various experimental wound models such excision model [9,10,12,15,21,26,28] incision model [10,12,21], burn wound model [7,12,20,21] and dead space wound model [10,12] has been used for evaluating various wound healing potentials of the polyherbal formulations under study. The excision model was prevalent in most of the studies. Moreover, rodents were used majorly as experimental model to study wound healing. Most of the studies evaluated the healing potential by determining one or a combination of the following attributes *viz.* wound contraction, collagen synthesis, wound size reduction, rate of wound closure and epithelialization. Ulcers in diabetic animal models were one of the key themes of study that could be identified in the review, owing to wound healing being a major problem in diabetic patients.

The second theme analyzed for wound healing is the anti inflammatory potential of polyherbal formulations. The healing process involves the release of pro-inflammatory cytokines from macrophages during the healing process. This upregulation leads to an increase in inflammatory reactions causing pain. In the post-inflammatory phase, anti-inflammatory agents suppress these cytokinins thereby, reducing the inflammatory response and accelerating the healing process [72]. However, in some circumstances, a prolonged inflammatory phase slows the wound healing process [73]. Therefore, wound management should ideally include an effective anti-inflammatory formulation to accelerate healing. The active compounds present in the polyherbal formulations, such as flavonoids and phenols are often useful in suppressing the production of inflammatory cytokines and thereby blocking the inflammatory transduction cascade [13]. These polyherbal formulations are reported to have natural anti-inflammatory activity with the lowest degree of side effects [44,48,52]. The literature review indicates various in-vivo studies depicting the potential of polyherbal formulations to suppress pro-inflammatory cytokinins and interleukins, thereby aiding in the wound-healing.

The third theme that has been addressed in the current review is an essential aspect of polyherbal formulations. With the growing multidrug resistance, using different antibiotics to control infections is a matter of grave concern [74]. Thus disciplines such as ethano-botany and ethano-pharmacology have been prominently researching validating the use of traditional medicines as anti-infectious agents [13]. This has a direct implication for wound healing because a delay in the healing process caused by underlying issues often makes an open wound a growing bed for microbes slowing down the healing process and making the wound more painful [75]. Foot ulcers in diabetic patients are an example of this. Therefore the current review has analyzed studies associated with evaluatiing the antimicrobial potentials of various wound care polyherbal formulations. In this context, it was observed that polyherbal formulations are effective against a good range of gram-positive bacteria, gram-negative bacteria, and fungi that are often associated with wound infection [55]. Some formulations are also derived for combating infections in areas prone to infectious wounds such as the vagina [56]. Many studies have mentioned that the antimicrobial nature of polyherbal formulations is also attributed to the phenol and flavonoid contents of the herbs [55,60].

Apart from this, it has also been observed that in most of the in vivo studies, there need to be more proper controls. This makes it even more challenging to come to a firm judgement. Therefore, using of appropriate study designs is essential to understand the actual potentials of polyherbal formulations. Furthermore, most of the in vivo studies focused on evaluating the potential of the selected polyherbal formulation on different wound types but no other variable was tested in this context. Therefore designing multivariate in vivo studies with variable treatment durations, treatment dose, type of infections could be an effective way to appropriately ascertain the effectively of any polyherbal formulation. Another important aspect that needs urgent attention is the toxicity assessment of the polyherbal formulations. The literature review depicts very few studies focusing on determining the toxic threshold of potent polyherbal wound healing formulations. Thus, toxicity assessment could be another important direction for future research on this theme.

## 5 Conclusion and future outlook

Medicinal plants contain several natural bio-active compounds with therapeutic potency used to synthesize several drug formulations. The multifaceted benefits of polyherbal formulations have been a focus of extensive research, but a systematic apporach is required to harness their full potential.. However, considering the key points from the literature research, it is evident that polyherbal preparations are highly effective in promoting wound healing. They have the potential to initiate a number of physiological functions that accelerate the process of wound healing. Therefore, these formulations merit further study in clinical trials, and production upscaling and removing the aforementioned bottlenecks will contribute to opening up a new horizon for polyherbal wound healing products that are more effective and have fewer side effects.

## Source of funding

None.

## Author contributions

**Ashwini Kumar Dixit (AKD):** AKD is responsible for the conception of the review paper as well as its supervision and editing.

**Satish Dubey (SD):** SD contributed with the analysis of themes, the gathering of data, and the literature bibliography.

Finally, after reviewing the article, both AKD and SD gave their approval to the final manuscript.

## Declaration of Competing Interest

None.

## References

[1] National Institutes of Health (2005).

[2] Mathew L., Babu S. (2011). Phytotherapy in India: transition of tradition to technology. Curr Bot.

[3] Srivastava S., Lal V.K., Pant K.K. (2013). Polyherbal formulations based on Indian medicinal plants as antidiabetic phytotherapeutics. Phytopharmacology.

[4] Spinella M. (2002). The importance of pharmacological synergy in psychoactive herbal medicines. Alternative Med Rev.

[5] Chorgade M.S. (2007). Drug discovery and development. Drug Dev.

[6] Webster J., Watson R.T. (2002). Analyzing the past to prepare for the future: writing a literature review. MIS Q.

[7] Khan I., Manzoor Z., Raza M.A., Munawar S., Manzoor A., Lodhi A. (2020). Assessment of ameliorative effect of Aab-e-Shifa polyherbal formulation in experimentally-induced wound in rabbits. Trop J Pharmaceut Res.

[8] Khan R.G., Jumaanand A., Taliab A. (2020). Formulation and evaluation of derma heal cream against wound and burn healing activity in streptozotocin-induced diabetic Wistar albino rat. Int J Pharma Sci Res.

[9] Malarvizhi R., Abhishek P., Barathidasan R., Kishore K.K., Vasanthi H. (2020). Potential wound healing properties of a polyherbal formulation (amrit oil) in an experimental model on wistar rats. Biomedicine.

[10] Jain P.K., Pounikar Y., Khurana N. (2020). Wound healing activity of aerial parts of Euphorbia hirta Linn. and its polyherbal formulation. Indian Drugs.

[11] Ponrasu T., Cheng T., Wang L., Cheng Y., Wang H.D. (2020). Natural biocompatible polymer-based polyherbal compound gel for rapid wound contraction and promote re-epithelialization: an in vivo study. Mater Lett.

[12] Majumder P., Paridhavi M.A. (2019). Novel Poly-herbal Formulation Hastens Diabetic Wound Healing with Potent Antioxidant Potential: A Comprehensive Pharmacological Investigation. Pharmacogn J.

[13] Dev S.K., Choudhury P.K., Srivastava R., Sharma M. (2019). Antimicrobial, anti-inflammatory and wound healing activity of polyherbal formulation. Biomed Pharmacother.

[14] Malviya K., Chouksey R. (2019). Exploration of wound healing activity of polyherbal formulation. J Drug Deliv Ther.

[15] Talekar Y.P., Apte K.G., Paygude S.V., Tondare P.R., Parab P.B. (2017). Studies on wound healing potential of polyherbal formulation using in vitro and in vivo assays. J Ayurveda Integr Med.

[16] Kadhiravan M., Keerthana K., Shobana G., Jothi G., Radhika J. (2017). Healing potential of a polyherbal ointment on excision wound in normal and diabetes-induced albino rats. Asian J Pharmaceut Clin Res.

[17] Hayat Z., Chaudhry M.A. (2017). Evaluation of a poly-herbal preparation for the treatment of peptic ulcer. Bangladesh J Pharmacol.

[18] Memariani Z., Hajimahmoodi M., Minaee B., Khodagholi F., Yans A., Rahimi R. (2017). Protective effect of a polyherbal traditional formula consisting of Rosa damascene mill., Glycyrrhiza glabra L. and Nardostachys jatamansi DC., against ethanol-induced gastric ulcer. Iran J Pharm Res (IJPR).

[19] Lee K.P., Kim J., Kim H., Chang H.R., Lee D.W., Park W. (2016). Bo-gan-whan regulates proliferation and migration of vascular smooth muscle cells. BMC Compl Alternative Med.

[20] Fahimi S., Mortazavi S., Abdollahi M., Hajimehdipoor H. (2016). Formulation of a traditionally used polyherbal product for burn healing and HPTLC fingerprinting of its phenolic contents. Iran J Pharm Res (IJPR).

[21] Aslam M.S., Ahmad M.S., Mamat A.S., Ahmad M.Z., Salam F. (2016). Antioxidant and wound healing activity of polyherbal fractions of Clinacanthus nutans and Elephantopus scaber. Evid Based Complement Alternat Med.

[22] Nehete M.N., Nipanikar S., Kanjilal A.S., Kanjilal S., Tatke P.A. (2016). Comparative efficacy of two polyherbal creams with framycetinsulfate on diabetic wound model in rats. J Ayurveda Integr Med.

[23] Kumari K.N., Venkateswarulu M., Suresh Babu M., DivyaSree M.S., Pravallika P. (2016). Evaluation of antiulcer potential of polyherbal preparation against experimentally induced ulcers in rats. Int J Pharmacogn Phytochem Res.

[24] Gaspar A., Savin S., Toma A. (2015). Phenolic content, antioxidant activity and effect on collagen synthesis of a traditional wound healing polyherbal formula. Studia Univ. VG, SSV (Life Sciences Series).

[25] Alexandru V., Gaspar A., Savin S., Toma A., Tatia R., Gille E. (2015). Phenolic content, antioxidant activity and effect on collagen synthesis of a traditional wound healing polyherbal formula. Studia Univ VG, SSV.

[26] Rajendren S., Muthuirulappan S. (2014). Wound healing activity of a polyherbal Siddha formulation. Int J Pharmtech Res.

[27] Ashok P., Gupta N., Murthy N., Shukla R., Rohita Sri G. (2014). Comparative wound healing activity of two polyherbal formulations GC-01 and GC-02 on experimentally induced wounds in rodents. Indian J Pharm Edu Res.

[28] Thube S.A., Patil M.J. (2014). Evaluation of wound healing potential of some Indian herbal extracts and it's formulation in acne vulgaris. Pharm J.

[29] Babu P.N., Nagaraju B., Vinay Kumar I. (2014). Evaluation of antiulcer and in-vitro antioxidant activities of Ixora coccinea flowers and polyherbal extract in wistar albino rats. Int J Pharm Pharmaceut Sci.

[30] Maheshwari R., Balaraman R., Sailor G., Parmar G., Patel A., Seth A.K. (2014). Antiulcer and antioxidant effects of normacid syrup (a polyherbal formulation) on experimentally-induced gastric ulcers. Orient Pharm Exp Med.

[31] Patra K.C., Jayaram Kumar K., Ahirwar D.K. (2014). Gastroprotective effect of standardized extract of amukkarachoornam on experimental gastric ulcer in rats. J Nat Med.

[32] Devaraj V.C., Krishna B. (2013). Antiulcer activity of a polyherbal formulation (PHF) from Indian medicinal plants. Chin J Nat Med.

[33] Purohit A.P., Kushwaha R. (2013). Antiulcer activity of polyherbal formulation. Int J Pharma Bio Sci.

[34] Fedekar F.M., Mohsen H., Waleed K., Walied A. (2013). Wound healing activity of Brown algae plus polyherbal extract in normal and alloxan-induced diabetic rats. J Adv Vet Res.

[35] Goyal S., Sureka S., Daniel K., Trivedi R., Deshmukh P., Navin S. (2012). Evaluation of wound healing effect of a polyherbal formulation by different cutaneous wound models. Pharmacologyonline.

[36] Balaji G., Ramesh B., Mahendranath G., Jagadeesh Kumar D., VenuBabu V. (2012). Protective effects of a poly herbal formulation against aspirin induced ulcers in wistar rats. Int J Pharm Pharmaceut Sci.

[37] Dash G., Murthy Pn (2011). The wound healing effects of a new polyherbal formulation. Der Pharm Lett.

[38] Papanas N., Maltezos E. (2011). Polyherbal formulation as a therapeutic option to improve wound healing in the diabetic foot. Indian J Med Res.

[39] Viswanathan V., Kesavan R., Kavitha K.V., Kumpatla S. (2011). A pilot study on the effects of a polyherbal formulation cream on diabetic foot ulcers. Indian J Med Res.

[40] Shanthi A., Radha R., Jaysree N. (2011). Antiulcer activity of newly formulated herbal capsule. Asian J Pharmaceut Clin Res.

[41] Nair V., Arjuman A., Gopalakrishna H.N., Dorababu P., Mirshad P.V., Bhargavan D. (2010). Evaluation of the anti-ulcer activity of NR-ANX-C (a polyherbal formulation) in aspirin & pyloric ligature induced gastric ulcers in albino rats. Indian J Med Res.

[42] Ilamathi K., Kumar R., Duraivel M., Prabhu D., Stanley S., Ruckmani A. (2020). Evaluation of anti-inflammatory activity of polyherbal formulation Vipro^TM^ in Lipopolysaccharide (LPS) induced inflammation in Wistar albino rats. Ann Trop Med Publ Health.

[43] Dadoriya P., Dey Y., Sharma D., Yadav M., Wanjari M., Gaidhani S. (2020). In-vitro anti-inflammatory and antioxidant activity of an ayurvedic formulation –Trayodashangguggulu. J Herb Med.

[44] Soujanya K., Reddy K., Kumaraswamy D., Reddy G., Girija P., Sirisha K. (2020). Evaluation of wound healing and antiinflammatory activities of new poly-herbal formulations. Indian J Pharmaceut Sci.

[45] Balkrishna A., Ranjan R., Sakat S.S., Sharma V., Shukla R., Joshi K. (2019). Evaluation of polyherbal ayurvedic formulation ‘peedantak Vati’ for anti-inflammatory and analgesic properties. J Ethnopharmacol.

[46] Jargalsaikhan B., Ganbaatar N., Urtnasan M., Uranbileg N., Begzsuren D. (2019). Anti-inflammatory effect of polyherbal formulation (PHF) on Carrageenan and lipopolysaccharide-induced acute inflammation in rats. Biomed Pharmacol J.

[47] Mohan M.C., Abhimannue A.P., Kumar B.P. (2018). Modulation of proinflammatory cytokines and enzymes by polyherbal formulation Guggulutiktakaghritam. J Ayurveda Integr Med.

[48] Al-Sayed E., Gad H.A., El-Shazly M., Abdel-Daim M.M., Nasser Singab A. (2018). Anti-inflammatory and analgesic activities of cupressu flavone from Cupressus macrocarpa: impact on pro-inflammatory mediators. Drug Dev Res.

[49] Ganga B., Wadud A., Jahan N., Shaikh A.A.M. (2018). Anti-inflammatory and analgesic activity of HabbeGuleAakh, A polyherbal Unani formulation in animal models. J Ayurveda Integr Med.

[50] Lee M., Su Hong, Park C., HanMin-Ho, Kim S., Hong S. (2017). Anti-inflammatory effects of Daehwangmokdantang, a traditional herbal formulation, in lipopolysaccharide-stimulated RAW 264.7 macrophages. Exp Ther Med.

[51] Nagarkar B., Jagtap S. (2017). Effect of new polyherbal formulations DF1911, DF2112 and DF2813 on CFA induced inflammation in rat model. BMC Compl Alternative Med.

[52] Dey D., Chaskar S., Athavale N., Chitre D. (2015). Acute and chronic toxicity, Cytochrome P450 enzyme inhibition, and hERG channel blockade studies with a polyherbal, ayurvedic formulation for inflammation. BioMed Res Int.

[53] Shrikhande P.V. (2013). Formulation and evaluation of polyherbal topical anti-inflammatory emulgel. Res J Pharm Technol.

[54] Velpandian T., Gupta P., Ravi A.K., Sharma H.P., Biswas N.R. (2013). Evaluation of pharmacological activities and assessment of intraocular penetration of an ayurvedic polyherbal eye drop (Itone™) in experimental models. BMC Compl Alternative Med.

[55] Sanpinit S., Yincharoen K., Jindamanee C., Jobthin S., Limsuwan S., Kunworarath N. (2020). Antibacterial properties of Ya-Samarn-Phlae (YaSP): a pilot study on diabetic patients with chronic ulcers. J Herb Med.

[56] Mishra N.N., Agarwal A., Moitra T., Polachira S.K., Nair R., Gupta S.K. (2019). Anti-HIV-1 activity and safety profile of a polyherbal gel formulation as a candidate microbicide. J Herb Med.

[57] Merish S., Walter T.M. (2019). Antimicrobial activity and Escherichia coli biofilm destruction potency of Siddha formulation Sagadevinei. Indian J Tradit Knowl.

[58] Patel P., Joshi C., Kothari V. (2019). Antipathogenic potential of a polyherbal wound-care formulation (Herboheal) against Certain wound-infective gram-negative bacteria. Adv Pharmacol Sci.

[59] Rajalakshmi K., Shanmugapriya P., Christian G.J., Gladys J., Banumathi V., Geethalakshmi S. (2018). Antimicrobial potential of Siddha polyherbal formulation AavaraiKudineer. J Pure Appl Microbiol.

[60] Patel P., Joshi C., Kothari V. (2018). Anti-pathogenic efficacy of a polyherbal wound-care formulation (Herboheal) against Staphylococcus aureus, and identifying its molecular targets. Infect Disord: Drug Targets.

[61] Okonkwo U., DiPietro L. (2017). Diabetes and wound angiogenesis. Int J Mol Sci.

[62] Icli B., Nabzdyk C.S., Lujan-Hernandez J., Cahill M., Auster M.E., Wara A.K.M. (2016). Regulation of impaired angiogenesis in diabetic dermal wound healing by microRNA-26a. J Mol Cell Cardiol.

[63] Nuutila K., Katayama S., Vuola J., Kankuri E. (2014). Human wound-healing research: issues and perspectives for studies using wide-scale analytic platforms. Adv Wound Care.

[64] Lawrence R., Tripathi P., Jeyakumar E. (2009). Isolation, purification and evaluation of antibacterial agents from Aloe vera. Braz J Microbiol.

[65] Knott A., Reuschlein K., Mielke H., Wensorra U., Mummert C., Koop U. (2008). Natural Arctium lappa fruit extract improves the clinical signs of aging skin. J Cosmet Dermatol.

[66] Tam J.C., Ko C.H., Lau K.M., To M.H., Kwok H.F., Chan Y.W. (2014). A Chinese 2-herb formula (NF3) promotes hindlimb ischemia-induced neovascularization and wound healing of diabetic rats. J Diabetes Complicat.

[67] Mi J., Wu C., Li C., Xi F., Wu Z., Chen W. (2014; 2). Two new triterpenoids from ampelopsis japonica (thunb.) Makino. Nat Prod Res.

[68] Nicolaus C., Junghanns S., Hartmann A., Murillo R., Ganzera M., Merfort I. (2017 Jan 20). In vitro studies to evaluate the wound healing properties of Calendula officinalis extracts. J Ethnopharmacol.

[69] Suntar I., Kupeli A.E., Nahar L., Satyajit D., Sarker S.D. (2012). Wound healing and antioxidant properties: do they coexist in plants. Free Radic Antioxidants.

[70] Geethalakshmi R., Sakravarthi C., Kritika T., Arul Kirubakaran M., Sarada D.V.L. (2013). Evaluation of antioxidant and wound healing potentials of Sphaeranthus amaranthoides Burm. f.. Biomed Res Int.

[71] Aslam M.S., Ahmad M.S., Mamat A.S. (2016). Phytochemical evaluation of polyherbal formulation of Clinacanthus nutans and Elephantopus scaber to identify flavonoids. Pharm J.

[72] Sharma A.K., Sunder V., Yashavarddhan M.H., Shukla S.K. (2017). Wound healing: current understanding and future prospect. Int J Drug Discov.

[73] Shukla S.K., Sharma A.K., Gupta V., Yashavarddhan M.H. (2019). Pharmacological control of inflammation in wound healing. J Tissue Viability.

[74] Joshi C., Patel P., Palep H., Kothari V. (2019). Validation of the anti-infective potential of a polyherbal ‘Panchvalkal’preparation, and elucidation of the molecular basis underlining its efficacy against Pseudomonas aeruginosa. BMC Compl Alternative Med.

[75] Negut I., Grumezescu V., Grumezescu A.M. (2018). Treatment Strategies for infected wounds. Molecules.

